# False-Positive Head-Impulse Test in Cerebellar Ataxia

**DOI:** 10.3389/fneur.2012.00162

**Published:** 2012-11-12

**Authors:** Olympia Kremmyda, Hanni Kirchner, Stefan Glasauer, Thomas Brandt, Klaus Jahn, Michael Strupp

**Affiliations:** ^1^Department of Neurology, University Hospital of MunichMunich, Germany; ^2^German Centre for Vertigo and Balance Disorders, University of MunichMunich, Germany; ^3^Bernstein Centre for Computational NeuroscienceMunich, Germany; ^4^Institute of Clinical Neurosciences, University of MunichMunich, Germany

**Keywords:** cerebellum, vestibuloocular reflex, head-impulse test, cerebellar atrophy, flocculus

## Abstract

The objective of this study was to compare the findings of the bedside head-impulse test (HIT), passive head rotation gain, and caloric irrigation in patients with cerebellar ataxia (CA). In 16 patients with CA and bilaterally pathological bedside HIT, vestibuloocular reflex (VOR) gains were measured during HIT and passive head rotation by scleral search coil technique. Eight of the patients had pathologically reduced caloric responsiveness, while the other eight had normal caloric responses. Those with normal calorics showed a slightly reduced HIT gain (mean ± SD: 0.73 ± 0.15). In those with pathological calorics, gains 80 and 100 ms after the HIT as well as the passive rotation VOR gains were significantly lower. The corrective saccade after head turn occurred earlier in patients with pathological calorics (111 ± 62 ms after onset of the HIT) than in those with normal calorics (191 ± 17 ms, *p* = 0.0064). We identified two groups of patients with CA: those with an isolated moderate HIT deficit only, probably due to floccular dysfunction, and those with combined HIT, passive rotation, and caloric deficit, probably due to a peripheral vestibular deficit. From a clinical point of view, these results show that the bedside HIT alone can be false-positive for establishing a diagnosis of a bilateral peripheral vestibular deficit in patients with CA.

## Introduction

The head-impulse test (HIT; Halmagyi and Curthoys, 1988) is the most important clinical bedside examination for the angular horizontal vestibuloocular reflex (VOR). It is performed by quick head thrusts, while the patient is asked to fixate a target, usually the nose of the examiner. If the VOR is intact, both eyes stay on target during and after the head rotation. If the VOR is impaired, the eyes move along with the head, and the patient has to make a re-fixation saccade to bring the eyes back on target. The HIT can be used to test the VOR at high and middle frequencies, whereas bithermal caloric irrigation tests the angular horizontal VOR at a low frequency which was calculated down to 0.003 Hz (Formby and Robinson, 2000). In the routine clinical praxis, a pathological HIT is therefore interpreted as a clinical sign of a peripheral vestibulopathy.

Several studies have shown an interrelationship between impaired function of the VOR and cerebellar disease, in particular in cerebellar patients with ocular motor syndromes, such as downbeat nystagmus. On the one hand, bilateral vestibular failure is often associated with cerebellar ataxia (CA) and cerebellar ocular motor signs (Rinne et al., 1998; Zingler et al., 2007). On the other hand, about 1/3 of patients with a downbeat nystagmus, which is most often caused by bilateral cerebellar floccular dysfunction, also show signs of an impaired VOR, with either a pathological HIT or pathological caloric irrigation, or both (Wagner et al., 2008). Further, the combination of CA, bilateral vestibular loss, and sensory polyneuropathy has been repeatedly described (Migliaccio et al., 2004; Zingler et al., 2007; Kirchner et al., 2011) and named CANVAS (CA with Neuropathy and Bilateral Vestibular Areflexia Syndrome; Szmulewicz et al., 2011a,b).

In order to identify the origin of VOR deficits in cerebellar patients (central vs. peripheral), we compared the results of the bedside HIT and the caloric irrigation, two classical peripheral vestibular tests, with objective measurements of the VOR gain in these patients.

## Materials and Methods

### Patients

#### Inclusion criteria

This prospective study included patients with gait and limb ataxia, cerebellar ocular motor signs (i.e., hypermetric saccades, impaired visual fixation suppression of the VOR, saccadic smooth pursuit, gaze-evoked nystagmus, and/or downbeat nystagmus), and a bilaterally pathological bedside HIT as tested by three experienced clinicians (Olympia Kremmyda, Klaus Jahn, and Michael Strupp). None of the patients had a positive family history for CAs. Genetic search for spinoCAs 1, 2, 3, and 6, which was performed in five patients, was negative. Voxel based morphometry MRI data from these patients showed cerebellar atrophy in all patients (Kirchner et al., 2011).

#### Exclusion criteria

Patients who had a history of exposure to aminoglycosides, Menière’s disease, meningitis, or other known causes of bilateral vestibulopathy according to a study on its etiology (Zingler et al., 2007) were excluded.

Written informed consent was obtained from all patients. The study was approved by the Ethics Committee of the Medical Faculty of the University of Munich.

#### Clinical examination

All patients received a complete standardized neurological, neuro-ophthalmological, and neuro-otological examination as described previously (Zingler et al., 2007).

##### Electromyography and neurography

Standard motor and sensory nerve conduction measurements (including medial, ulnar, peroneal, tibial, and sural nerve) were performed for the diagnosis of a polyneuropathy. An electromyography of the *M. tibialis* anterior was also obtained from all patients but three, who were under phenprocoumon therapy. Axonal neuropathy was defined by a reduction of nerve conduction amplitudes and demyelinating neuropathy by reduction of nerve conduction velocities. If both amplitude and velocity was reduced we classified the neuropathy as mixed. Normative nerve conduction amplitude and velocity values were taken from age matched tables from our neurology department (Kirchner et al., 2011).

#### Caloric irrigation

Caloric testing was performed with bithermal caloric irrigation (water temperature 30° and 44°, duration of irrigation 30 s) and with ice water (0°). Caloric-induced nystagmus was recorded by means of electronystagmography using the Igor Pro Wave Metric Software (Version 3.13 Wave Metrics, Inc., Lake Oregon) or by video-oculography using the EyeSeeCam system (www.eyeseecam.com). A peak slow phase velocity of the caloric-induced nystagmus of less than 5 deg/s in both ears was considered reduced responsiveness (Vesterhauge and Kildegaard Larsen, 1977).

### Eye and head movement recordings

Eye movements were recorded using dual search coils (Skalar, Delft, The Netherlands) that were placed on the left eye after local anesthesia with oxybuprocaine 4%. Head movements were measured using two custom-made coils mounted on a head ring with an angle of 90 deg between them. Subjects were seated in the center of a cubical magnetic field made of 140-cm-long bars (Remmel, MA, USA). The calibration method is described in detail in Glasauer et al. (2003). The patients were tested on the following paradigms:

#### Horizontal head-impulses (HIT)

Centrifugal, short head-impulses to both sides that amounted to up to 30 deg were given to each patient while they were fixating a central target (distance 140 cm) in order to evaluate the horizontal VOR at high frequency. Only those impulses that reached a peak velocity of 75 deg/s and a peak acceleration of 1000 deg/s^2^ in the first 150 ms were included in this study. Further inclusion criteria applied were as follows: the head velocity at movement onset had to be below 20 deg/s; no change of sign of the relevant component of the head velocity had occurred during the impulse; and the rotation axis of the peak velocity had to be within 45 deg of the intended (horizontal) direction (Glasauer et al., 2004).

#### Smooth pursuit

Subjects had to follow a laser dot that moved sinusoidally with a frequency of 0.167 Hz vertically (±17 deg) and horizontally (±17 deg) without moving their heads (peak velocity 17.8 deg/s). The smooth pursuit gain could not be calculated in one patient due to a large coil slip.

#### Passive rotation VOR and visual fixation suppression of the VOR

The patient was asked to fixate a target that was generated from a laser mounted on the head ring (head-fixed target), while his/her head was being manually rotated at a 0.33-Hz frequency, maximal velocity 40 deg/s (VORsupp). Then the patient was instructed to fixate an imaginary central target in the dark while the examiner rotated his/her head at a frequency of 0.33 Hz (VORdark). Finally, a central target was switched on and the patient was asked to keep fixating it while his/her head was being rotated (VORlight). VOR gains (defined as eye velocity/head velocity) were computed for all patients under the different conditions.

### Statistics

Statistics and data analysis were performed in MatLab (The MathWorks) using *t*-test for comparing average values. For correlations, Pearson correlation was used. Statistical significance for rejecting the null hypothesis of equal means between two groups was defined as *p* < 0.05.

## Results

### Patients

**Figure 1:**
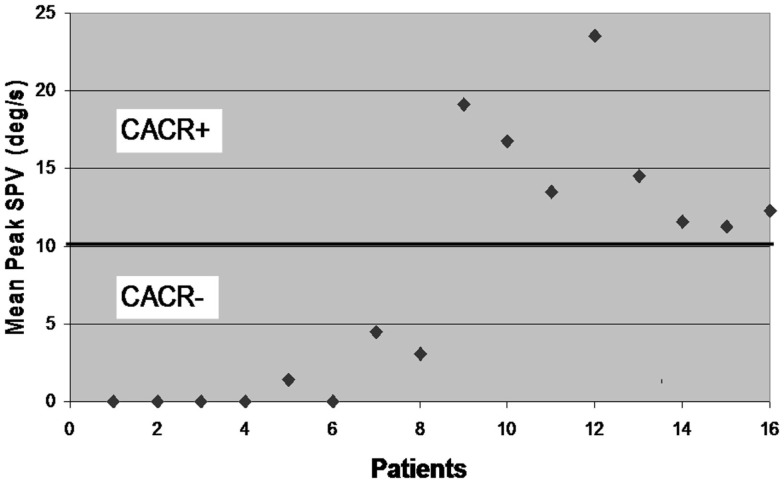
**Mean peak slow phase velocity (SPV) for each of the 16 patients included in the study**. According to the results of the caloric irrigation, the cerebellar patients are divided into two groups: patients 1–8 with mean peak SPV < 5 deg/s (CACR−) and patients 9–16 with mean peak SPV > 10 deg/s (CACR+).

Sixteen patients (Male: 12) participated in the study. Eight of them had a pathological caloric response (mean peak slow phase velocity ± SD of the bithermal irrigation (SPV): 1.1 ± 1.8 deg/s; Figure 1). In what follows, we refer to this as the CACR− group. Five of these eight CACR− patients had no caloric response, also in the ice water caloric test. The remaining eight patients, who will be referred as the CACR+ group, had normal caloric responses (SPV > 10 deg/s, mean peak SPV: 15.3 ± 4.3 deg/s).

### Clinical features

The CACR− patients were on average 73.1 ± 9.3 years old, the CACR+ patients were 69.8 ± 5.7 years old (*p* = 0.359). The mean age at the beginning of the symptoms was 62.8 ± 9.7 years in the CACR− group and 60.6 ± 3.8 years in the CACR+ group (*p* = 0.573). The mean disease duration in the CACR− and in the CACR+ group was 10.4 and 9.1 years, respectively (*p* = 0.387).

The main symptom of both groups was an unsteady gait: all patients reported a worsening of gait imbalance on uneven ground. Oscillopsia during walking or head movements was reported by six of the eight CACR− patients and by four of the eight CACR+ patients. Four of the CACR− and three of the CACR+ patients had a downbeat nystagmus, one CACR+ patient an upbeat nystagmus. All patients had a saccadic smooth pursuit, two CACR− patients only vertically. All CACR− patients had clinical signs of a polyneuropathy in the lower extremities (reduced vibration sense and missing ankle reflex), whereas only half of CACR+ patients had a clinical polyneuropathy. Nerve conduction studies and electromyography, showed polyneuropathy in 4/8 CACR+ patients and in 6/8 CACR− patients, mostly of mixed sensory/motor type. An overview of the clinical findings is given in Table 1.

**Table 1:** **Ocular motor and electromyographic findings in patients with normal (CACR+) and reduced/absent (CACR−) caloric response**.

Patient	Age	EMG/NCS				Clinical examination					
		clPNP	PNP	Mot./sens.	Ax/dem	HIT R	HIT L	SVV	SP	SPN	GEN
CACR− 1	80	1	0	0	0	Path.	Path.	0	Vert.	0	Hor
CACR− 2	58	1	1	3	3	Path.	Path.	L	All dir.	Downbeat	Hor/down
CACR− 3	80	1	1	3	1	Path.	Path.	0	All dir.	0	Hor
CACR− 4	70	1	1	3	3	Path.	Path.	0	All dir.	0	Hor
CACR− 5	66	1	0	0	0	Path.	Path.	n.e.	Down	Downbeat	None
CACR− 6	71	1	1	3	3	Path.	Path.	R	All dir.	0	Hor/down
CACR− 7	80	1	1	3	1	Path.	Path.	L	All dir.	Downbeat	Hor/down
CACR− 8	80	1	1	3	3	Path.	Path.	0	All dir.	Downbeat	Hor/down
CACR+ 1	76	1	1	1	3	Path.	Path.	0	All dir.	Downbeat	Hor/down
CACR+ 2	63	0	0	0	0	Path.	Path.	0	All dir.	0	Hor/down
CACR+ 3	70	1	1	3	3	Path.	Path.	L	All dir.	Upbeat	Hor/up
CACR+ 4	66	0	0	0	0	Path.	Path.	R	All dir.	0	Hor/down
CACR+ 5	67	0	0	0	0	Path.	Path.	n.e.	All dir.	Downbeat	None
CACR+ 6	80	0	0	0	0	Path.	Path.	0	All dir.	0	Hor
CACR+ 7	66	1	1	3	3	Path.	Path.	0	All dir.	Downbeat	Hor/down
CACR+ 8	70	1	1	1	3	Path.	Path.	0	All dir.	0	Hor/down

*clPNP, clinical diagnosis of polyneuropathy (missing deep tendon reflexes and reduced vibration sensation). PNP, polyneuropathy according to electroneurographic criteria. Mot/sens, 1: motor, 2: sensory, 3: mixed neuropathy; ax/dem, 1: axonal, 2: demyelinating, 3: mixed neuropathy. HIT, head-impulse test; SVV, subjective visual vertical; SP, smooth pursuit; SPN, spontaneous nystagmus; GEN, gaze-evoked nystagmus; n.e., not examined; R, right; L, left*.

### Eye movement recordings

#### HIT gain measured by the scleral coil technique

**Figure 2:**
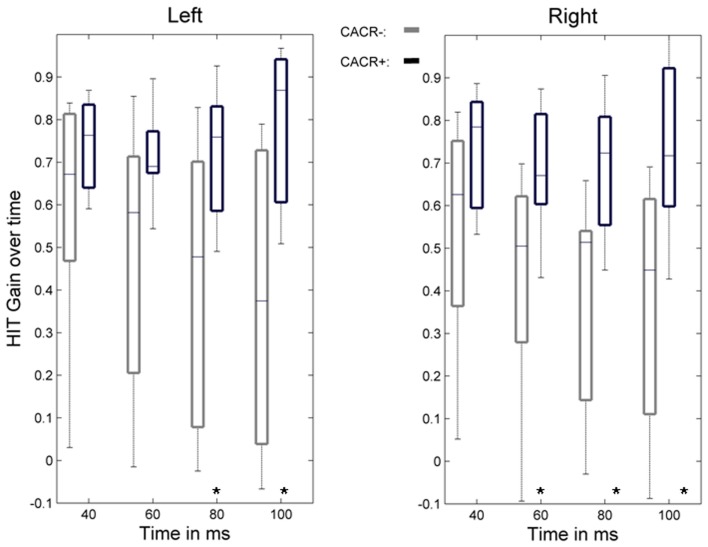
**Time course of the VOR gain during the head-impulse test**. Boxplots show the VOR gain measured by the scleral coil technique at 40, 60, 80, and 100 ms for left- and rightward head-impulses for the group of patients with normal caloric response (CACR+) and reduced/absent caloric response (CACR−). Boxplot whiskers indicate the data range, the middle line the median value, and the edges of the boxes the upper and lower quartile. Although the initial VOR gain at 40 ms did not statistically differ between the two groups, gain values at 80 and 100 ms were statistically higher in the CACR+ group. Statistically significant values are marked with (*), for *p* values see text.

**Figure 3:**
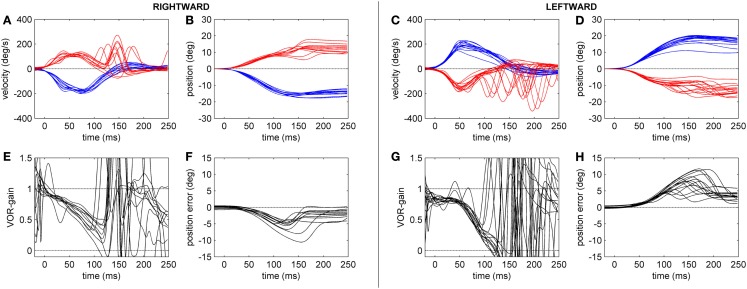
**Head-impulse test recordings of a CACR− patient**. Subplots **(A,B,E,F)** refer to rightward and subplots **(C,D,G,H)** to leftward HIT data. Subplots **(A,C)** show eye (red tracks) and head (blue tracks) velocity (deg/s) data for the first 250 ms after starting the head-impulse. Subplots **(B,D)** show the eye and head position (in deg) over time. Subplots **(E,G)** show the time course of the HIT gain (eye/head velocity). Subplots **(F,H)** show the position of the eye in space (in deg), here defined as position error (ideal value zero).

**Figure 4:**
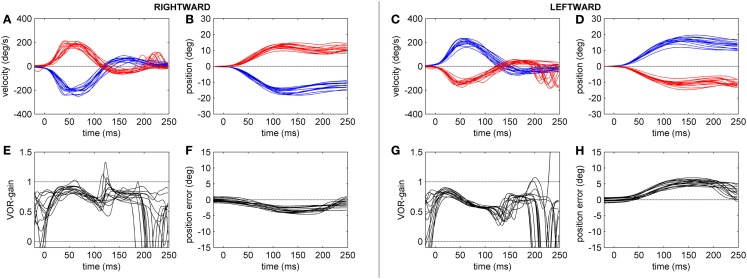
**Head-impulse test recordings of a CACR+ patient**. Subplots **(A,B,E,F)** refer to rightward and subplots **(C,D,G,H)** to leftward HIT data. Subplots **(A,C)** show eye (red tracks) and head (blue tracks) velocity (deg/s) data for the first 250 ms after starting the head-impulse. Subplots **(B,D)** show the eye and head position (in deg) over time. Subplots **(E,G)** show the time course of the HIT gain (eye/head velocity). Subplots **(F,H)** show the position of the eye in space (in deg), here defined as position error (ideal value zero).

The gain of the angular, horizontal VOR (eye velocity/head velocity) was quantified by the HIT and eye and head velocities were measured by the scleral coil technique at 40, 60, 80, and 100 ms after the head-impulse was initiated (Figure 2). The data were compared between the two groups. Figures 3 and 4 show HIT recordings from a CACR− (Figure 3) and CACR+ (Figure 4) patient. Group analysis revealed no statistical significant difference between the two groups at 40 ms (average gain CACR−: 0.597 ± 0.279 (mean ± SD) for leftward HITs, 0.545 ± 0.279 for rightward HITs, CACR+: 0.742 ± 0.108 and 0.733 ± 0.138 respectively, *p* > 0.1 between groups). VOR gain in the CACR− group for rightward HIT was significantly lower at 60 ms than in the CACR+ group (0.426 vs. 0.685 respectively, *p* = 0.034) but not for leftward HIT (0.48 vs. 0.71, *p* = 0.068). At 80 and 100 ms the difference between the gains in the two groups was statistically significant for both sides (80 ms: CACR− leftward: 0.415 ± 0.332, rightward: 0.378 ± 0.252, CACR+: 0.721 ± 0.152 and 0.691 ± 0.164, *p* = 0.033 and 0.011 between the groups for each side respectively, 100 ms: CACR− leftward: 0.376 ± 0.338, rightward: 0.369 ± 0.296, CACR+: 0.789 ± 0.185 and 0.738 ± 0.207, *p* = 0.008 and 0.013 between the groups for each side). These values indicate that the HIT gain decreased from 40 to 100 ms in the CACR− group, whereas in the CACR+ it remained unchanged (Figure 2).

##### Corrective saccades after HIT

**Figure 5:**
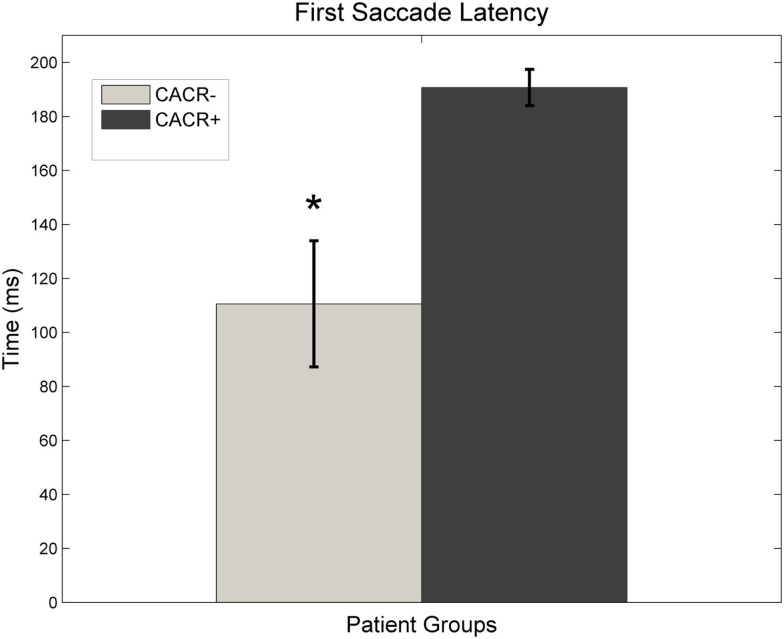
**Average latency of the first saccade made after initiation of the head-impulse for both groups**. Data for right and left head-impulse are pooled. CACR+ patients make their first saccade almost constantly at about 200 ms (after the head movement is over), whereas CACR− patients make their first corrective saccade much earlier, often much before the head movement is over.

The CACR− patients made their first corrective saccade on average at 110 ± 62 ms (Figure 5), whereas CACR+ patients made their first corrective saccade much later (on average at 191 ± 17 ms, *p* = 0.0064) after HIT onset. The latency of the first saccade for the CACR− patients correlated with the gain of the VOR at 100 ms (two-tailed Pearson correlation: *R* = 0.883, *p* = 0.008 for rightward HIT and *R* = 0.785, *p* = 0.036 for leftward HIT). This shows that the lower the gain in the first 100 ms, the earlier a corrective saccade occurred. Such a correlation was not observed in the CACR+ group (two-tailed Pearson correlation: *R* = 0.376, *p* = 0.462 for rightward HIT and *R* = 0.562, *p* = 0.190 for leftward HIT).

#### Angular VOR in the dark and visual fixation suppression of the VOR

**Figure 6:**
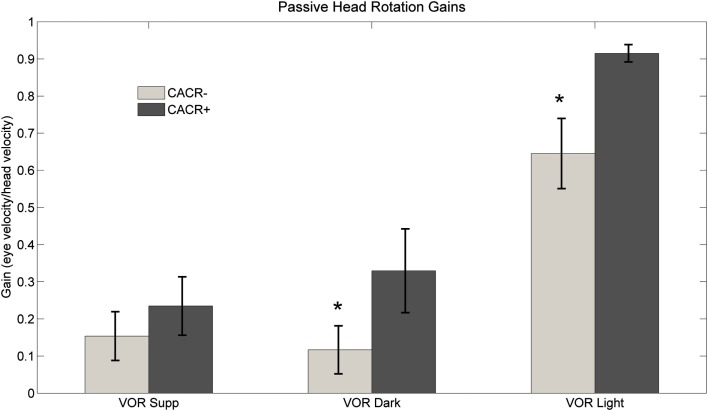
**Average VOR suppression, VOR dark, and VOR light gain at 0.33 Hz for both groups**. Although VOR suppression gains did not differ between the two groups, gains for middle frequency VOR in dark and in light were higher in the CACR+ than in the CACR− group, similar to the HIT gains VOR gains. Asterisks (*) indicate statistical significance between the two patient groups, error bars represent standard error of the mean.

CACR− patients showed reduced VOR gains in the dark compared to CACR+ (VOR dark: 0.12 ± 0.19 vs. 0.42 ± 0.2, *p* = 0.0075) and while fixating the central target (VOR light: CACR−: 0.65 ± 0.27 vs. CACR+: 0.92 ± 0.07, *p* = 0.0105; Figure 3B). Visual fixation suppression of the VOR was not significantly different between the two groups (*p* = 0.442), although the gain was lower in the CACR− group (VOR Supp CACR−: 0.15 ± 0.17 vs. CACR+: 0.23 ± 0.23; Figure 6).

##### Smooth pursuit

There was no statistically significant difference in the smooth pursuit gains (eye velocity/target velocity) in all directions between the two groups. Smooth pursuit gains were as follows: down: 0.21 ± 0.19 (CACR−), 0.33 ± 0.19 (CACR+), *p* = 0.24; up: 0.38 ± 0.20 (CACR−), 0.55 ± 0.21 (CACR+), *p* = 0.14; left: 0.40 ± 0.32 (CACR−), 0.58 ± 0.22 (CACR+), *p* = 0.234; right: 0.41 ± 0.31 (CACR−) 0.64 ± 0.21 (CACR+), *p* = 0.128. Horizontal smooth pursuit gains were positively correlated with VOR suppression gains (one-tailed Pearson correlation), both in the CACR− group (*R* = 0.706, *p* = 0.0025) and in the CACR+ group (*R* = 0.698, *p* = 0.0407).

## Discussion

The two major aims of this clinical study were as follows: First, to evaluate the discrepancies between the results of the bedside HIT and of the caloric irrigation in cerebellar patients and second to gain further insight into the pathophysiology of a reduced VOR gain associated with CA by comparing the results of the HIT, passive rotation, and calorics in these patients. With respect to the caloric response, the cerebellar patients could be divided into two groups: patients with a pathological caloric response (CACR− group), and patients with a normal caloric response (CACR+ group).

Quantitative measurements of the VOR gain with the scleral coil technique showed that in the CACR− patients the VOR gain at 60, 80, and 100 ms after the initiation of the HIT decreased during the HIT. The CACR+ group showed a moderately reduced VOR gain, which remained stable during the HIT.

CACR− patients also showed reduced VOR gains during passive head rotation in darkness and in the presence of a target in comparison to the CACR+ patients. Normally, during passive head rotation in light (VORlight), gaze stabilization (target fixation) can be achieved either through smooth pursuit or VOR pathways. This means, even if one of the two pathways is lesioned, a normal gain could be achieved anyway. In cerebellar patients with peripheral vestibular lesions, both pathways are lesioned, therefore, one would expect lower VORlight gain values. In an earlier study (Migliaccio et al., 2004), it was suggested that patients with bilateral vestibulopathy and cerebellar disease have pathological values during slower passive head rotation while fixating a target because of a combined vestibular and smooth pursuit deficit, which was the case for the CACR− patients, but not for the CACR+ patients who had a high gain in this test(mean 0.92).

Is it then possible that the reduced HIT gain in the CACR+ patients is the result of a central (cerebellar), and not a peripheral vestibular lesion? There are several anatomical connections between the vestibular nuclei and the cerebellum, in particular the vestibulocerebellum, i.e., flocculus, paraflocculus, nodulus, and uvula. The floccular complex modulates the VOR through these polysynaptic, anatomical connections (Rambold et al., 2002). Mossy fibers from the vestibular nuclei end bilaterally in the nodulus and part of the uvula. Fibers from the vestibular nuclei and nucleus prepositus hypoglossi project directly to the flocculus (Buttner-Ennever, 1992). Conversely, Purkinje cells from the flocculus and nodulus directly project to the vestibular nuclei (Nieuwenhuys et al., 1991).

Earlier studies (Crane et al., 2000) have shown that patients with CA with normal calorics have reduced VOR gains. In non-human primates it was shown that whereas lesions of the flocculus lead to a mild-to-moderate decrease of the VOR gain at high frequencies (Lisberger et al., 1984), they either increase, decrease, or have no influence on VOR gain at low frequencies (Zee et al., 1981). This correlates with our data showing an isolated high frequency VOR gain deficit in the CACR+ group. Accordingly these patients showed floccular atrophy in the voxel base morphometry (Kirchner et al., 2011).

All CACR− patients began making corrective saccades before the end of the head movement (covert saccades); a similar pattern was observed in patients with bilateral vestibulopathy due to gentamicin ototoxicity (Weber et al., 2009). This earlier saccade could be triggered from the decreasing VOR gain during the HIT. The latency of the first saccade correlated with the VOR gain; the lower the gain, the earlier the saccade. CACR+ patients did not make any corrective saccades during the HIT, only about 200 ms after the beginning of the HIT (overt saccades), probably because the VOR gain remained stable during the HIT. In contrast to patients with peripheral vestibulopathy, no correlation between VOR gain and first saccade latency was observed at these patients.

Could this pathological HIT in the CACR+ patients simply result from a peripheral vestibular deficit limited in the high frequencies, in an earlier stage of the disease? Patients with CA and peripheral vestibulopathy (CANVAS), who have the same oculomotor findings as our CACR− patients (Migliaccio et al., 2004), show histopathologically atrophy of the vestibular nerves (Szmulewicz et al., 2011a). CACR+ patients, although they have statistically the same age and disease duration as the CACR− ones (one CACR+ patient had dizziness symptoms since 20 years), have normal calorics, a finding which contradicts the argument of an ongoing peripheral vestibular atrophy.

Taking the above into account, we conclude that the measurements of the HIT and passive rotation VOR gain with the scleral coil technique and the caloric results do not support a bilateral peripheral vestibular deficit diagnosis in the CACR+ patients, which might have arisen from the clinical observation of a pathological HIT. In other words, our data show that the bedside HIT is less reliable in patients with CA. These patients therefore have a false-positive HIT.

In conclusion, we identified two groups of patients with CA and pathological HIT: one (CACR−) with a HIT, passive rotation, and caloric VOR deficit, most likely due to a combined degeneration of the vestibular system and the cerebellum, as clinical, caloric, and MRI data show (Kirchner et al., 2011). The other group (CACR+) had a moderate isolated high-acceleration VOR deficit, most likely due a central cerebellar flocculus dysfunction. Clinically, therefore, these patients have a pathological bedside HIT, which might not indicate a peripheral vestibular dysfunction (a “false-positive” HIT). Our study underlines the importance of additional vestibular testing in patients with CA in order to establish a diagnosis of bilateral vestibulopathy.

## Conflict of Interest Statement

The authors declare that the research was conducted in the absence of any commercial or financial relationships that could be construed as a potential conflict of interest.
